# The Neuroscience Community Has a Role in Environmental Conservation

**DOI:** 10.1523/ENEURO.0454-20.2021

**Published:** 2021-03-17

**Authors:** Joyce Keifer, Cliff H. Summers

**Affiliations:** 1 Neuroscience Group, Basic Biomedical Sciences, Sanford School of Medicine, University of South Dakota, Vermillion, SD 57069; 2 Department of Biology, University of South Dakota, Vermillion, SD 57069; 3 Veterans Affairs Research Service, Sioux Falls VA Health Care System, Sioux Falls, SD 57105

**Keywords:** biodiversity, comparative neuroscience, conservation, human health, neuroscience policy, sustainability

## Abstract

We previously argued that the neuroscience community has a role in environmental conservation because protection of biodiversity and the specialized behavioral adaptions of animals is essential to understanding brain structure and function. Preserving biodiversity and the natural world is also linked to human mental health and broadens our insight on the origins of psychiatric disorders like stress, anxiety, and depression. The study of neuroscience has become a global scientific pursuit that involves thousands of researchers and has an economic impact in the billions of dollars. As a group of biomedical research scientists, neuroscientists have the knowledge base and public credibility to convincingly promote sustainable environmental actions and policies. Here, we outline several key areas in which we as a neuroscience academic community can participate to preserve a rich global biodiversity and confront the environmental crises that lie before us.

## Significance Statement

Biodiversity and the global environment are currently undergoing unprecedented threats stemming from climate change and other sources of environmental stress that are rapidly leading toward widespread habitat loss and species extinction. These events endanger not only plant and animal species but human health and well-being. Environmental conservation limits habitat degradation that leads to disease from pollution, brain injury through neurotoxicity, and mental illness. Therefore, the neuroscience community has a direct stake in working for the protection and preservation of a rich global environmental biodiversity. By promoting sustainable actions, policies, and approaches to biomedical research, neuroscientists can and should have a leadership role in developing strategies that will benefit the environment and human health.

## Introduction

In a recent startling report released by the Intergovernmental Science-Policy Platform on Biodiversity and Ecosystem Services (IPBES; May, 2019; https://www.ipbes.net/), it was estimated that at least 1 million plant and animal species are threatened by global extinction. The main catalysts of this calamitous situation are loss/degradation of habitat in land and sea, unsustainable exploitation of animals and plants, climate change resulting from increased greenhouse gas emissions, pollution, and invasive species. Many scientists believe that in this “Anthropocene epoch,” humanity is approaching the sixth mass extinction in the history of Earth, the last one being ∼65 million years ago (Barnosky et al., 2011; Ceballos et al., 2017). The IPBES study concluded that the loss of biodiversity globally is not only an environmental issue, but also an economic, social, and moral one.

In an earlier paper (Keifer and Summers, 2016), we argued that it was important for the neuroscience community to play a significant role in environmental conservation. This is because conservation efforts preserve the unique habitats that drive the emergence of specialized behavioral adaptations and their underlying neural circuitry across diverse species. Scientific investigation into the substrates of brain function and behavior has benefitted immeasurably from comparative studies of the anatomy and physiology of brains from different species (Bingman, 1992; Manger, 2011; Striedter et al., 2014; Enard, 2016; Keifer and Summers, 2016; van den Heuvel et al., 2016). Further, many of the tools currently in use for neuroscience research have been developed from a diversity of organisms, such as clinically-useful toxins/venoms (e.g., to treat cancer or channelopathies), use of bacterial light-sensitive ion channels for optogenetics, fluorescent proteins such as GFP, and CRISPR gene editing (Fenno et al., 2011; Kelsh et al., 2011; Schendel et al., 2019). A comparative approach helps neuroscience researchers identify conserved features of neural systems across animal groups that reveal fundamental genetic and physiological principals of brain structure and function. Moreover, preservation of biodiversity and the natural environment can be linked to human feelings of well-being and mental health (Bratman et al., 2015, 2019; Cabrera et al., 2016). Recognition of the interdependence of humans and nature (Comberti et al., 2015) broadens our insight into the origins of psychiatric disorders such as stress, anxiety and depression (Carr and Summers, 2002; Blanchard et al., 2013; Bratman et al., 2015). Ultimately, examining human health in the broader context of the environment translates to a deeper understanding of brain function and the etiology and treatment of human psychiatric disease.

In the face of rapidly growing environmental crises, the neuroscience community can take a leadership role in protecting and preserving a rich global environmental biodiversity. The Society for Neuroscience (SFN), the flagship scientific society for neuroscientists, has >37,000 members worldwide and annual expenditures of over $30 million. Moreover, neuroscience research is allocated ∼$7 billion from the National Institutes of Health (NIH) each year in the United States alone. In addition to its economic impact, as a group of biomedical research scientists, we have the knowledge base and public credibility to convincingly promote sustainable environmental actions and policies. Neuroscientists also have a direct stake in preserving biodiversity both from a brain research perspective and as advocates for mental health. In this article, we outline several key areas in which we as individual scientists or as an academic community can participate to promote conservation and associated goals through modified approaches to the neuroscience research endeavor in concert with awareness of the global environment.

## What We Can Do as a Neuroscience Community

### Leverage research funding to promote sustainable environmental impacts by corporations

Research funding, particularly in the form of grants, used to purchase equipment and supplies by institutions and their biomedical research laboratories can be a significant force for leveraging corporations to operate in environmentally conscientious ways. Approximately $80 billion is spent annually on basic research in the United States. How and where those funds are spent has the attention of corporate CEOs, local governments, and lawmakers. Consumer purchasing shifts the ways corporations produce and distribute goods. One example is the rapid rise in organic food production that has increased widely in recent years because of demand for healthier products by consumers. Similarly, selective purchasing from manufacturers of environmentally responsible goods and services by universities and individual research labs will drive economic markets toward positive change. To this aim, the University of California System adopted a detailed Policy on Sustainable Practices in 2019 (https://www.ucop.edu/sustainability/policy-areas/index.html). This policy describes system-wide goals and guidelines in nine areas including clean building design and sustainable transportation, foodservices, energy, and water usage. Sustainable procurement was highlighted because purchasing decisions that reduce negative impacts on the environment should have priority. Many other universities have followed suit and have established large sustainability initiatives. Smaller sustainability programs promoted by student groups have also begun to make an impact. Such programs in North America are tracked and rated (the green score) by the Association for the Advancement of Sustainability in Higher Education (https://www.aashe.org/). These efforts will have an important impact on reducing potentially harmful consequences of biomedical research and other campus activities on the environment. Individual neuroscience laboratories can also take initiatives to purchase environmentally friendly products where possible. For example, academic consumers can inform sales representatives that they are interested in purchasing goods made from and shipped using recyclable and biodegradable materials. Moreover, researchers can request that items be packaged and shipped together to use less packing material and consumption of fuel to deliver them. This “sustainable procurement and consumption” approach by universities and neuroscience research labs will have the overall effect of diminishing the environmental impact of doing biomedical research. While these initiatives are a good start to increasing sustainability, the challenge is in making these alternatives systemic to the marketplace. If efforts to conserve resources are time consuming and laborious, they will fail. A case in point is, again, organic goods. Some years ago, such products were of limited availability and difficult to find. But increased public awareness of the health and environmental benefits combined with responsive farmers and manufacturers stimulated substantial growth of this industry (Mie et al., 2017; Demirtas, 2019). Today, organic products and foods are now abundant and acquired easily in most stores (although the growth of this movement was aided by an act of Congress to establish national standards). If the past is instructive, then the persistence of the academic consumer and their purchasing power will drive industrious suppliers to respond with more environmentally friendly products and services. Making these sustainable practices a systemically marketplace-driven reality would be facilitated by the participation of large scientific societies such as the SFN and governmental agencies like the NIH that have important roles in supporting biomedical research.

### Decrease overconsumption by neuroscience laboratories

The impact of overconsumption of laboratory animals, supplies, services, water and energy and the subsequent generation of biomedical waste by the medical field contributes to a severe impact on the environment (Manzoor and Sharma, 2019). These activities combine to result in global climate change, habitat destruction, toxic pollution from hazardous materials, and decline in species and biodiversity. Neuroscience research contributes to a relatively small percentage of the waste generated compared with hospitals and other health care facilities, but it is hardly inconsequential. Neuroscience researchers are increasingly concerned about the impact of their labs on the local community and are looking for ways to make their work more ecologically sustainable. Along with the sustainability initiatives described above, changes in standard lab practices can lead to “green labs” (Webb, 2016; Gross, 2018; Zimmer, 2018; Zak et al., 2020). Many lab procedures are performed from entrenched practices and habit. Examples include constantly changing pipette tips or unnecessarily using new plastic PCR tubes. Cutting down on consumables, especially disposable single-use plastics, not only saves the lab money but reduces the amount of waste that goes into landfills. Simple ideas like lowering the set temperature of ultra-low freezers from −80°C to −70°C significantly reduces the energy used without being detrimental to samples. Closing chemical fume hoods reduces energy usage and costs. This has led to “Shut the Sash” initiatives at many universities. Powering down computers and other equipment not in use would also contribute to energy savings. Since individual researchers do not directly pay the energy usage bills of their institutions, one idea is to post energy consumption data outside of labs. This might encourage greater frugality and friendly competition among labs to conserve, especially if there was a system of reward. Along these lines, standard autoclaves use ∼50 gallons of water per minute when in use. Far more efficient instruments (e.g., Water-Mizer) are available that reduce water usage by half and should be considered when autoclaves need replacement. There are a number of online resources for researchers to consult to help make their labs greener and reduce their environmental impact (Webb, 2016).

### Reduce travel to neuroscience conferences

Global aviation contributes 2.4% of all carbon dioxide (CO_2_) emissions from fossil fuels. This amount would have ranked sixth compared with nations and was the largest source of CO_2_ from energy producers in 2015 (Graver et al., 2020). Accounting for contrails and other greenhouse gas emissions from air travel pushes that figure higher. The environmental impact of travel is becoming an increasing concern among the scientific research community and there is an expanding willingness to explore new options (Pulizzi, 2019; Geitmann, 2020; Haage, 2020). One possibility is for departments to host more seminar speakers from nearby institutions. This option has the added benefit of potentially increasing collaborations. Currently, the arrival of the COVID-19 pandemic and ensuing cancellations of meetings and reluctance to travel on the part of attendees, particularly by plane, has resulted in the wide use of alternatives to in-person attendance at conferences and other speaking events. Neuroscientists should push large scientific societies like the SFN and those in Europe represented by the Federation of European Neuroscience Societies (FENS) toward providing remote access to their annual meetings. New platforms increase the available options for videoconferencing through Internet services. Notably, the FENS Forum 2020 meeting was held entirely online and was accessible worldwide. Smaller meetings, workshops, and departmental seminars could be augmented or hosted by resources such as Zoom (https://www.zoom.us/), which is now commonly used in academics because of the pandemic. This would allow departments to reduce the costs of seminars and host more speakers. The NIH has also experimented with holding Internet and video assisted study section meetings. The effectiveness of this approach, however, is somewhat controversial and is still being evaluated. Although virtual seminars are not likely to completely replace scientific meetings, use of these resources can give neuroscientists a choice to limit travel in the future and significantly reduce the carbon footprint of individual researchers for the long term (Geitmann, 2020). Another significant point with regard to remote meeting access is that it will substantially enhance the equity and inclusion of speakers and attendees. Scientists who have difficulty or are reluctant to travel because of child care, expense, disability, scheduling, or a variety of other reasons, could more easily participate in study sections, give seminars, and attend meetings if they are held online. Remote access would also be global, opening the door to investigators in economically challenged countries or remote locations. Implementation and expanded use of remote viewing could be a major benefit to neuroscience research overall. More detailed recommendations for enhancing sustainable travel behavior can be found elsewhere (Haage, 2020). One benefit of reducing time-consuming long-haul travel is that the time spent away from labs and students will decline, thereby increasing overall productivity and the quality of home life for individuals.

### Advance public policies for lawmakers that promote environmental conservation and human health

Strategies that contribute significantly to sustainability efforts and changes in environmental policy that benefit the environment and human health should be advanced by neuroscience societies and organizations. Environmental degradation impacts human health in numerous ways including malnutrition and injury as a result of natural disasters such as drought and flooding that have intensified, spread of vector-borne diseases because of global warming, and toxicity leading to chronic diseases from air and water pollution (Myers et al., 2013; Rossati, 2017; Morens and Fauci, 2020). Far less attention is given to mental health and well-being (Cabrera et al., 2016; Bratman et al., 2019), problems that are just as pervasive and personally devastating. Moreover, the cultural worldviews of indigenous peoples and their place in nature factors into loss of feelings of self-worth, stability and wellness when local ecosystems decline. Scientific neuroscience societies like the SFN can significantly impact policy development to promote a healthier environment for wildlife and humans. Currently, most of the advocacy efforts of the SFN, FENS, and other groups are focused on promoting the advancement of neuroscience and research funding targeting brain injury and disease. In our view, however, this is a missed opportunity. Neuroscience societies should be more actively engaged in supporting environmental justice, preservation, and sustainability to create a safer, less toxic, less stressful world. Importantly, positive change in resolving these concerns will result in a reduction of neurologic and mental health burdens. Along these lines, environmental sustainability adds economic value and jobs related to the changes required from current practice such that economic well-being associated with these efforts has positive effects on mental health. These economic changes may be simple, such as converting conventionally plowed fields to no-till farming. This results in reduction of legacy CO_2_ concentration in the atmosphere, improves sustainability, regenerates soil quality, and provides greater profit for farmers while reducing the environmental load of toxic chemicals (Rainbow and Derpsch, 2011; Osei et al., 2012). Better economic outcomes, especially in combination with environmental improvement, surely promotes improved mental health. Further, there could be targeted efforts to address specific opportunities or concerns raised by neuroscience research to drive political policy changes that improve the environment and the human condition. Issues, for example, like the effects of environmental neurotoxins on brain development (such as the lead water crisis that occurred in Flint, MI, in 2014 and is ongoing; Ruchart et al., 2019), or sonar interference by military operations on animal navigation (naval sonar interference resulting mass strandings of whales; Tyack et al., 2011; Goldbogen et al., 2013; Morell, 2015), should be addressed using scientific data and potential solutions offered by experts. These groups could also strengthen their effectiveness by partnering with other scientific (American Association for the Advancement of Science, AAAS) and worldwide organizations (the Intergovernmental Panel on Climate Change of the UN) to enhance public awareness and advance informed positions on significant global issues such as climate change. Ultimately, the advocacy of scientific societies is driven by the membership they represent. Members who are also scientific experts create an important additional political force for influencing legislators that there is significant scientific consensus for new laws related to climate change, environmental and wildlife conservation, and reduction of toxic pollutants, as well as the health and well-being issues that environmental problems promote.

### Outlook for the future

We have unexpectedly learned from the COVID-19 pandemic that some of the negative impacts of human activities on Earth can be reversible. Satellite imaging revealed that during the time of global quarantine starting in early 2020, carbon emissions dramatically declined and the atmosphere became clearer and cleaner worldwide, particularly over urban areas (Le Quere et al., 2020; Earth Observing Dashboard, https://www.eodashboard.org/). Anecdotal reports indicate that wildlife activity near towns and cities normally reduced during the day to avoid humans has increased and taken on more natural patterns (Rutz et al., 2020). Although these positive changes must be sustained to have a lasting global impact on the environment, the current COVID-19 crisis is instructive. It offers a temporary worldwide laboratory to examine the effects of human activity on our planet that points research, and some inventive thinking, toward significant steps that can be taken to reduce our impact. While these positive changes give us hope, they do not suggest that the severe damage done to the Earth’s environment can be rectified quickly. Global climate change is promoted by feedback loops between the atmosphere and biomass that further support increased CO_2_ levels and temperature (van Nes et al., 2015; Curran and Curran, 2016; Williams et al., 2019). These complex interactions are just beginning to be understood and it is thought they can become self-sustaining if human activity is not changed. It is possible that we are near, or have already passed, the threshold for this carbon-cycle feedback system in forcing climate change. The time to act is now. Reversing the environmental damage caused by human populations to the Earth will require transformational economic and social change. The neuroscience research community can help lead the way for other professional scientific organizations to leverage their knowledge, authority and will to bring about significant change that addresses the global crises that confront us.

## Synthesis

Reviewing Editor: Christophe Bernard, INSERM & Institut de Neurosciences des Systèmes Decisions are customarily a result of the Reviewing Editor and the peer reviewers coming together and discussing their recommendations until a consensus is reached. When revisions are invited, a fact-based synthesis statement explaining their decision and outlining what is needed to prepare a revision will be listed below.

The authors havee addressed the remaining concerns

## Author Response

Dear Dr. Bernard,

Thank you for informing us that our manuscript, "Neuroscience has a Role in Environmental Conservation" could be acceptable for publication in eNeuro with some revisions. We have incorporated all of the comments of the reviewers into the text and these changes are highlighted in red font. They are listed below:

1. This type of paper must be focused on one message. The section "Expand International Resources for Specimens and Databases Available for Neuroscience Research" is a different topic and needs to removed.

In response, this section was removed from the paper along with the references.

2. Please, change also the title. Neuroscience cannot have a role. Neuroscientists can.

We have changed the title to "The Neuroscience Community has a Role in Environmental Conservation".

We hope that with the changes made to the manuscript in response to the reviews, the paper will now be acceptable for publication in eNeuro. We look forward to hearing from you.

Sincerely yours,

Joyce Keifer, Ph.D.

Cliff H. Summers, Ph.D
